# IGNITE4: Results of a Phase 3, Randomized, Multicenter, Prospective Trial of Eravacycline vs Meropenem in the Treatment of Complicated Intraabdominal Infections

**DOI:** 10.1093/cid/ciy1029

**Published:** 2018-12-18

**Authors:** Joseph S Solomkin, Janis Gardovskis, Kenneth Lawrence, Philippe Montravers, Angie Sway, David Evans, Larry Tsai

**Affiliations:** 1 Department of Surgery, University of Cincinnati College of Medicine, Ohio; 2 Department of Surgery, Riga Stradins University, Latvia; 3 Tetraphase Pharmaceuticals, Watertown, Massachusetts; 4 Département d’Anesthésie-Réanimation, CHU Bichat Claude Bernard; 5 Université Paris Diderot, PRESS Sorbonne Cité, Paris, France; 6 Institut National de la Santé et de la Recherche Médicale (INSERM) UMR, Paris, France; 7 World Surgical Infection Society, Cincinnati, Ohio; 8 Department of Surgery, Ohio State University School of Medicine, Columbus

**Keywords:** complicated intraabdominal infection, eravacycline, multidrug resistance, gram-negative bacteria, Enterobacteriaceae

## Abstract

**Background:**

Increasing antimicrobial resistance among pathogens that cause complicated intraabdominal infections (cIAIs) supports the development of new antimicrobials. Eravacycline, a novel member of the fluorocycline family, is active against multidrug-resistant bacteria including extended-spectrum β-lactamase (ESBL) and carbapenem-resistant Enterobacteriaceae.

**Methods:**

IGNITE4 was a prospective, randomized, double-blind trial. Hospitalized patients with cIAI received either eravacycline 1 mg/kg every 12 hours or meropenem 1 g every 8 hours intravenously for 4–14 days. The primary objective was to demonstrate statistical noninferiority (NI) in clinical cure rates at the test-of-cure visit (25–31 days from start of therapy) in the microbiological intent-to-treat population using a NI margin of 12.5%. Microbiological outcomes and safety were also evaluated.

**Results:**

Eravacycline was noninferior to meropenem in the primary endpoint (177/195 [90.8%] vs 187/205 [91.2%]; difference, –0.5%; 95% confidence interval [CI], –6.3 to 5.3), exceeding the prespecified margin. Secondary endpoints included clinical cure rates in the modified ITT population (231/250 [92.4%] vs 228/249 [91.6%]; difference, 0.8; 95% CI, –4.1, 5.8) and the clinically evaluable population (218/225 [96.9%] vs 222/231 [96.1%]; (difference, 0.8; 95% CI –2.9, 4.5). In patients with ESBL-producing Enterobacteriaceae, clinical cure rates were 87.5% (14/16) and 84.6% (11/13) in the eravacycline and meropenem groups, respectively. Eravacycline had relatively low rates of adverse events for a drug of this class, with less than 5%, 4%, and 3% of patients experiencing nausea, vomiting, and diarrhea, respectively.

**Conclusions:**

Treatment with eravacycline was noninferior to meropenem in adult patients with cIAI, including infections caused by resistant pathogens.

**Clinical Trials Registration:**

NCT01844856.

## Teaser

This phase 3 trial compared eravacycline to meropenem for treatment of complicated intraabdominal infections. Eravacycline was noninferior to meropenem. High rates of presumed microbiological eradication were found with both regimens.

Gram-positive and gram-negative organisms with novel antimicrobial resistance mechanisms have been highlighted as a pressing global public health threat by learned societies, the World Health Organization, and government agencies [1–4]. For gram-negative organisms, a common resistance mechanism is the production of extended-spectrum β-lactamases (ESBLs) that deactivate most penicillin and cephalosporin molecules [5]. Estimates of morbidity, mortality, and cost stemming from complications caused by these resistant organisms are striking [6, 7]. The emergence of carbapenem resistance in Enterobacteriaceae is a particularly significant problem, as carbapenems have been the standard first choice for treating infections due to ESBLs, with few currently available alternatives [8–12].

Global surveys have provided estimates for the extent of the problem [13, 14]. For ESBL-producing *Escherichia coli* and *Klebsiella pneumoniae* isolates in Europe, rates of approximately 15% and 30%, respectively, have been detected. In North America, rates are approximately 10% for both isolates [15], relatively low when compared to the 40% detected for ESBL-producing *E. coli* in Southeast and East Asia and 60%–70% in China [16]. Carbapenemase-producing pathogens are responsible for a rapidly increasing number of clinical infections in specific geographic regions, the result of both clonal expansion and transfer of carbapenemase genes through mobile genetic elements [17, 18]. Other important pathogens, such as *Enterococcus faecium* and *Acinetobacter baumannii*, are also routinely multidrug resistant [19, 20]. These troubling epidemiological findings define the need for novel agents active against these bacterial pathogens.

Complicated intraabdominal infections (cIAIs) are defined as consequences of perforations of the gastrointestinal tract that result in contamination of the peritoneal space. If not immediately dealt with, this further results in abscess formation, peritonitis, and sepsis syndromes [21]. They are common occurrences in clinical practice and result in considerable consumption of resources for healthcare facilities and morbidity and mortality in patients. The infecting flora are typically polymicrobial, involving synergistic interactions between gram-positive, gram-negative facultative, aerobic, and anaerobic organisms [21]. Early empiric initiation of antimicrobial therapy effective against the range of infecting pathogens, intended to serve as both prophylaxis for surgical site infections and as therapy for established invasive infections, is established practice and is recommended in current guidelines [22, 23]. These infections have been important in the investigation of new antimicrobials because the diseases encompassed by the term cIAI are acute, come to clinical attention rapidly, and require invasive procedures to control, affording a high likelihood of pathogen identification.

Eravacycline is a novel, fully synthetic fluorocycline antibiotic developed for the treatment of serious infections, including cIAI, that inhibits bacterial protein synthesis by binding to the 30S ribosomal subunit [24]. It retains activity in the presence of common tetracycline-specific acquired-resistance mechanisms (ie, efflux, ribosomal protection) [25] and has potent in vitro activity against a broad range of susceptible and multidrug-resistant gram-positive and gram-negative aerobic and anaerobic strains, including *Staphylococcus aureus*, *E. faecium, E. coli*, *K. pneumoniae*, *A. baumannii*, and *Bacteroides* spp. [9, 26–28].

We previously performed a phase 2 study to provide an initial estimate of efficacy at the 2 highest dose regimens explored in normal volunteer studies [29, 30]. A total of 139 patients with confirmed cIAI were randomized (2:2:1) to receive eravacycline 1.5 mg/kg every 24 hours, eravacycline 1 mg/kg every 12 hours, or ertapenem 1 g every 24 hours, for a minimum of 4 days and a maximum of 14 days. The clinical success rates were greater than 90% in each arm. The incidence of treatment-emergent adverse events (TEAEs) was 35.8%, 28.6%, and 26.7%, respectively.

We then conducted a phase 3 trial wherein patients received eravacycline 1 mg/kg every 12 hours or ertapenem 1 g every 24 hours for a minimum of four 24-hour dosing cycles. For the microbiological intent-to-treat (micro-ITT) population, the rates of clinical cure at the test-of-cure (TOC) visit were 86.8% in the eravacycline group and 87.6% in the ertapenem group. The difference in clinical cure rates between the groups was −0.80% (95% confidence interval [CI], −7.1, 5.5), meeting the prespecified noninferiority (NI) margin of 10%.

The current phase 3 trial was undertaken to satisfy US Food and Drug Administration (FDA) requirements for a second trial in this indication. It provided the opportunity to examine the efficacy of eravacycline compared to another broad-spectrum carbapenem, meropenem.

## METHODS

### Study Design

This was a phase 3, randomized, double-blind, double-dummy, multicenter, prospective trial designed to test the safety and efficacy of eravacycline compared to meropenem in acutely hospitalized patients diagnosed with cIAI requiring operative or percutaneous intervention. Participants for this study were recruited from 65 sites in 11 countries.

The study protocol and all relevant supporting information were submitted to the institutional review board/independent ethics committee at each study site for approval prior to study initiation. The trial was conducted in accordance with Good Clinical Practice as described by the International Council for Harmonisation Guideline and consistent with the World Medical Assembly Declaration of Helsinki.

### Participants

Patients aged ≥18 years who were hospitalized for suspected cIAI and able to provide informed consent were considered for inclusion. Exclusion criteria included the following: considered unlikely to survive the 6- to 8-week study period; creatinine clearance <50 mL/min; presence or possible signs of significant hepatic disease; immunocompromised condition; history of hypersensitivity to tetracyclines, carbapenems, or beta-lactams; participation in any investigational drug or device study within 30 days of study entry; known or suspected nervous system disorder that suggests a predisposition to seizures; and receipt of effective antibacterial drug therapy for cIAI for more than 24 hours in the 72 hours prior to randomization. A complete listing of inclusion and exclusion criteria can be found in the Supplementary Materials.

### Sample Size

Estimations of cure rates and numbers of participants in the micro-ITT population came from the recent phase 3 study with eravacycline in cIAI (IGNITE1), in which ertapenem was used as the comparator. Using a 12.5% NI margin, 1-sided alpha of 0.025, 80% power, and response rates of 84% in the eravacycline treatment group and 85% in the meropenem treatment group, 161 participants per arm in the micro-ITT population were required. A sample size of approximately 466 randomized participants was then estimated to provide sufficient numbers for this study based upon an evaluability rate of 70%.

### Blinding and Randomization

Patients who met all of the inclusion criteria and none of the exclusion criteria were enrolled into the study and randomized using a computer-based randomization scheme. Randomization was stratified based on primary site of infection (complicated appendicitis vs all other cIAI diagnoses). The randomization process incorporated an enrollment cap of 50% for patients with complicated appendicitis.

A designated randomization administrator maintained the randomization codes in accordance with standard operating procedures to ensure that the blind was properly maintained. Only personnel who required knowledge of treatment assignments were unblinded.

### Intervention

The patients were enrolled and randomized to 1 of the 2 study arms: intravenous (IV) eravacycline (1 mg/kg every 12 hours) or IV meropenem (1 g every 8 hours). Due to the varying infusion volumes and times for the 2 study drugs, each patient received 5 infusions, all prepared by an unblinded pharmacist or designee. A duration of therapy of 4 to 14 complete dose cycles of the assigned drug was provided at the treating physician’s discretion. The expected duration of patient participation for the study was approximately 6–8 weeks. Treatment duration at study entry was expected to be a minimum of four 24-hour dosing cycles.

### Source Control Review

A single surgical reviewer (J. S. S.) examined the records of all patients considered clinical failures, or cures with an unplanned second procedure, or deaths. Source control was considered adequate when the physical measures at operation or drainage were consistent with current local standards of practice to drain infected fluid collections, eliminate the source of infection, control ongoing contamination, and restore gastrointestinal function [31]. Patients who were considered to have had inadequate source control were assigned indeterminate outcomes and were excluded from per-protocol analyses.

### Clinical Outcome Assessments and Statistical Analyses

The primary endpoint was the clinical response at the TOC visit 25–31 days after initiation of the study drug in the micro-ITT population, as required by the FDA. As eravacycline had demonstrated NI at a 10% NI margin in the IGNITE1 study, an NI margin of 12.5% was used in IGNITE4 as agreed to by the FDA. This is the standard margin for the Europeans Medicines Agency (EMA).

Secondary endpoints were clinical and microbiological responses for the micro-ITT, modified ITT, clinically evaluable, and microbiologically evaluable populations at end-of-treatment (EOT), TOC, and follow-up (FU) visits.

### Microbiological Specimen Collection and Outcome Assessments

Appropriate aerobic and anaerobic specimens for culture at the time of the on-study source control procedure were collected from the site of infection and directly inoculated into culture media during the procedure. Specimen collection was either by tissue biopsy or aspirate. These specimens were cultured, and the species were identified at a local or regional laboratory. All purified isolates were sent to the central reference laboratory for confirmation of species identification and antimicrobial susceptibility testing. Isolates were screened for possible ESBL or carbapenemase production based on antimicrobial susceptibility testing, which was confirmed by next-generation sequencing.

## RESULTS

**Figure 1.**
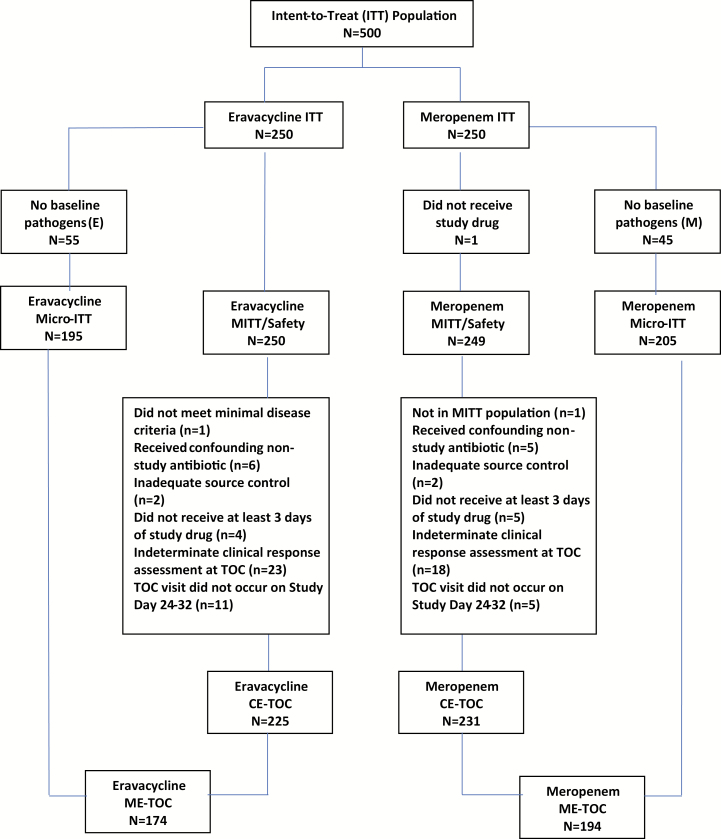
CONSORT diagram. Abbreviations: CE, clinically evaluable; ME, microbiologically evaluable; micro-ITT, microbiological intent-to-treat; MITT, modified intent-to-treat; TOC, test-of-cure.

A total of 500 patients were enrolled in the ITT population, 250 in each treatment arm. Figure 1 displays the CONSORT flow diagram. The majority of patients were enrolled in the European Union. Enrollment ran from 13 October 2016 to 1 April 1 2017.

Table 1 displays the demographics data for the micro-ITT population. The baseline demographics for patients in both treatment arms were similar. Sixty percent of patients in the eravacycline group and 63.4% in the meropenem group received open surgery; 35.4% and 32.7%, respectively, received laparoscopic surgery; and the remaining received percutaneous or other procedures. As randomized, 48.2% of the eravacycline group were diagnosed with complicated appendicitis vs 43.9% in the meropenem group.

**Table 1.** Demographics and Baseline Characteristics: Microbiological Intent-to-Treat Population

Characteristic	Eravacycline (N = 195)	Meropenem (N = 205)
Age, years		
Mean ± standard deviation (min., max.)	50.3 ± 17.7 (18, 84)	52.3 ± 18.3 (19, 87)
Age group, n (%)		
<65 years	148 (75.9)	145 (70.7)
65–75 years	34 (17.4)	38 (18.5)
>75 years	13 (6.7)	22 (10.7)
Gender, n (%)		
Female	86 (44.1)	100 (48.8)
Body mass index, kg/m^2^	27.4 ± 5.3 (17.2, 49.2)	27.1 ± 5.0 (17.1, 43.8)
Acute Physiology and Chronic Health Evaluation II score	6.6 ± 3.8 (0, 19)	6.4 ± 4.0 (0, 20)
Surgical intervention^a^		
Open	117 (60.0)	130 (63.4)
Laparoscopic	69 (35.4)	67 (32.7)
Percutaneous	12 (6.2)	15 (7.3)
Other	0 (0.0)	1 (0.5)

^a^In some cases, patients initially treated with laparoscopic or percutaneous therapy were converted to other procedures. Therefore, these categories are not mutually exclusive.

Details of the infections encountered are listed in Table 2. The majority of patients in both groups were enrolled post-operatively.

**Table 2.** Pathologies: Microbiological Intent-to-Treat Population

Pathology	Eravacycline (N = 195)	Meropenem (N = 205)
Actual primary disease diagnosis		
Complicated appendicitis, n (%)	94 (48.2)	90 (43.9)
Other complicated intra-abdominal infection	101 (51.8)	115 (56.1)
Diagnosed and enrolled preoperatively	7 (3.6)	11 (5.4)
Diagnosed intra-/postoperatively	188 (96.4)	194 (94.6)
Intra-abdominal abscess(es)^a^	119 (63.3)	110 (56.7)
Peritonitis	94 (50.0)	95 (49.0)
Gastric/duodenal perforation	11 (5.9)	12 (6.2)
Complicated cholecystitis	40 (21.3)	45 (23.2)
Perforation of small intestine	7 (3.7)	7 (3.6)
Complicated appendicitis	93 (49.5)	91 (46.9)
Perforation of large intestine	8 (4.3)	12 (6.2)
Diverticulitis with perforation, peritonitis, or abscess	5 (2.7)	7 (3.6)
Other	0	2 (1.0)

^a^The population included some patients with abscesses and no other diagnosis.

Table 3 shows the clinical response for all populations at the TOC visit. The primary efficacy endpoint, per FDA requirements, was the clinical response at the TOC visit in the micro-ITT population.

**Table 3.** Clinical Response at Test-of-cure Visit

Population	Eravacycline	Meropenem	Difference (95% Confidence Interval)
Modified intent-to-treat	N = 250	N = 249	…
Clinical cure	231 (92.4)	228 (91.6)	0.8 (–4.1, 5.8)
Clinical failure	7 (2.8)	9 (3.6)	…
Indeterminate/Missing	12 (4.8)	12 (4.8)	…
Microbiological intent-to-treat	N = 195	N = 205	…
Clinical cure	177 (90.8)	187 (91.2)	–0.5 (–6.3, 5.3)
Clinical failure	7 (3.6)	7 (3.4)	…
Indeterminate/Missing	11 (5.6)	11 (5.4)	…
Clinically evaluable	N = 225	N = 231	…
Clinical cure	218 (96.9)	222 (96.1)	0.8 (–2.9, 4.5)
Clinical failure	7 (3.1)	9 (3.9)	…
Indeterminate/Missing	0	0	…
Microbiologically evaluable	N = 174	N = 194	…
Clinical cure	167 (96.0)	187 (96.4)	–0.4 (–4.9, 3.8)
Clinical failure	7 (4.0)	7 (3.6)	…
Indeterminate/Missing	0	0	…

For this endpoint, the cure rate was 90.8% for eravacycline and 91.2% for meropenem, a difference of −0.5% with a 95% CI of −6.3% to 5.3%, meeting the predetermined criterion for NI. Clinical cure rates were high across all visits and populations, ranging from 90.8% to 96.9% in the eravacycline arm and from 91.2% to 96.4% in the meropenem arm. The percentages of patients with a response of clinical cure at the FU visit (Table 4) were similar between the treatment groups in all analysis populations and were generally lower than those at the EOT (Table 5) or TOC visits in all populations assessed. The latter observation was due to the higher number of missing responses in both treatment groups. Overall, the results for analysis of clinical cure were supportive of the primary efficacy analysis results.

**Table 4.** Clinical Response at Follow-up Visit

Population	Eravacycline (Clinical Cure/Total)	Meropenem (Clinical Cure/Total)	Difference (95% Confidence Interval)
Intent-to-treat	224/250 (89.6)	226/250 (90.4)	–0.8 (–6.2, 4.6)
Modified intent-to-treat	224/250 (89.6)	226/249 (90.8)	–1.2 (–6.5, 4.2)
Microbiological intent-to-treat	170/195 (87.2)	185/205 (90.2)	–3.1 (–9.5, 3.2)
Clinically evaluable	220/229 (96.1)	221/231 (95.7)	0.4 (–3.5, 4.3)
Microbiologically evaluable	168/177 (94.9)	184/192 (95.8)	–0.9 (–5.7, 3.6)

**Table 5.** Clinical Response at End-of-treatment Visit

Population	Eravacycline (Clinical Cure/Total)	Meropenem (Clinical Cure/Total)	Difference (95% Confidence Interval)
Intent-to-treat	235/250 (94.0)	234/250 (93.6)	0.4 (–4.0, 4.8)
Modified intent-to-treat	235/250 (94.0)	234/249 (94.0)	0.0 (–4.3, 4.4)
Microbiological intent-to-treat	181/195 (92.8)	193/205 (94.1)	–1.3 (–6.5, 3.7)
Clinically evaluable	232/239 (97.1)	234/237 (98.7)	–1.7 (–4.8, 1.1)
Microbiologically evaluable	180/187 (96.3)	193/196 (98.5)	–2.2 (–6.2, 1.2)

The microorganisms identified at the intraabdominal site of infection are detailed in Table 6. All patients in the micro-ITT population for both treatment arms had a baseline intraabdominal specimen, and only 1 patient did not have baseline blood culture samples. Almost all intraabdominal specimens (>99% of patients in both treatment arms) had confirmed bacterial growth in culture. Seven percent of blood cultures from both the eravacycline and the meropenem populations had confirmed growth. The risk of bacteremia regardless of treatment was highest with large or small bowel perforation (15% and 14.3%, respectively). Bacteremia in the micro-ITT population did not have an obvious effect on clinical outcome. Of the 29 such patients with bacteremia, 3 failed, 2 were indeterminant for missing endpoint visit, and 24 were cured. All patients with baseline bacteremia in the eravacycline group and all but 1 in the meropenem group had documented clearance of the baseline organism from the blood.

**Table 6.** Clinical Cure at the Test-of-cure Visit by Baseline Pathogen: Microbiological Intent-to-treat Population

Baseline Pathogen^a^	Eravacycline (N = 195)	Meropenem (N = 205)
Gram-negative aerobes	141/158 (89.2)	153/166 (92.2)
Enterobacteriaceae	129/146 (88.4)	142/154 (92.2)
*Escherichia coli*	111/126 (88.1)	125/134 (93.3)
*Klebsiella pneumoniae*	21/21 (100.0)	23/27 (85.2)
Non-enterobacteriaceae	36/38 (94.7)	28/30 (93.3)
*Acinetobacter baumannii* complex	5/5 (100.0)	2/2 (100.0)
*Pseudomonas aeruginosa*	18/19 (94.7)	18/20 (90.0)
Gram-positive aerobes	108/122 (88.5)	98/107 (91.6)
*Enterococcus avium*	10/11 (90.9)	9/10 (90.0)
*Enterococcus faecalis*	29/31 (93.5)	26/28 (92.9)
*Enterococcus faecium*	25/29 (86.2)	22/23 (95.7)
*Staphylococcus aureus*	16/16 (100.0)	7/8 (87.5)
Methicillin-susceptible *Staphylococcus aureus*	15/15 (100.0)	7/8 (87.5)
*Streptococcus* species	52/60 (86.7)	46/50 (92.0)
*Streptococcus viridans* group	50/57 (87.7)	40/44 (90.9)
*Streptococcus anginosus* group	39/45 (86.7)	31/33 (93.9)
*Streptococcus anginosus*	25/29 (86.2)	21/22 (95.5)
*Streptococcus constellatus*	13/15 (86.7)	9/11 (81.8)
*Streptococcus mitis* group	13/14 (92.9)	11/12 (91.7)
Anaerobes	99/110 (90.0)	104/111 (93.7)
*Bacteroides* species	83/94 (88.3)	82/88 (93.2)
*Bacteroides caccae*	5/6 (83.3)	5/5 (100.0)
*Bacteroides fragilis*	33/40 (82.5)	35/38 (92.1)
*Bacteroides ovatus*	19/24 (79.2)	28/28 (100.0)
*Bacteroides thetaiotaomicron*	27/30 (90.0)	30/33 (90.9)
*Bacteroides uniformis*	14/16 (87.5)	14/14 (100.0)
*Bacteroides vulgatus*	27/28 (96.4)	23/23 (100.0)
*Clostridium* species	9/9 (100.0)	26/26 (100.0)
*Clostridium perfringens*	7/7 (100.0)	12/12 (100.0)
*Fusobacterium* species	5/6 (83.3)	2/2 (100.0)

^a^Organisms encountered 10 or more times are included, along with those considered of interest. A full listing of baseline pathogens can be found in the Supplementary Materials.

A total of 284 patients (71%) had polymicrobial infection, and 320 patients harbored gram-negatives. *Bacteroides* species were found in 176 patients, and 158 of these also had at least 1 gram-negative. *Bacteroides* were cultured from only 1 patient with a monomicrobial infection.

We encountered a variety of ESBLs and carbapenem-resistant Enterobacteriaceae (CRE; see Table 7). The most common ESBL was CTX-M-15. One KPC-2 and 1 OXA-48 were encountered, both patients successfully treated with meropenem.

**Table 7.** Extended Spectrum Beta-lactamases

	Eravacycline (Cured/Total)	Meropenem (Cured/Total)
*Citrobacter freundii*	0	1/1
CTX-M-15	0	1/1
*Enterobacter cloacae/asburiae*	3/3	1/1
CTX-M-15	2/2	1/1
*Escherichia coli*	8/10	5/7
CTX-M-15	7/8	3/5
CTX-M-3	0/1	1/1
CTX-M-32	1/1	0
CTX-M-5	0	1/1
SHV-12	0	1/1
*Klebsiella pneumoniae*	5/5	5/6
CTX-M-15	5/5	3/4
CTX-M-2	0	1/1
SHV-12	0	1/1
*Serratia marcescens*	0	1/1
CTX-M-15	0	1/1

Organisms encountered often tested positive for more than 1 enzyme.

Clinical failures are detailed in Table 8. The primary reasons for failure in both groups were the need for an unplanned surgical or percutaneous procedure (5 in each group) and initiation of rescue antibiotic therapy for cIAI (6 in each group).

**Table 8.** Clinical Failure at Test-of-cure: Microbiological Intent-to-treat Population

Classification	Eravacycline (N = 195)	Meropenem (N = 205)
Clinical failure at test-of-cure	7^a^ (3.6)	7 (3.4)
Death due to cIAI	0	0
Persistence of clinical symptoms of cIAI	1 (14.3)	3 (42.9)
Unplanned surgical procedure or percutaneous drainage procedures for complication or recurrence of cIAI	5 (71.4)	5 (71.4)
Postsurgical wound infection requiring systemic antibiotics	2 (28.6)	0
Initiation of rescue antibacterial therapy for cIAI	6 (85.7)	6 (85.7)
Surgical Adjudication Committee correction^b^	0	1 (14.3)
Other	0	0

Abbreviation: cIAI, complicated intraabdominal infection.

^a^Some patients fell into multiple categories.

^b^This is defined as a patient who was classified as a clinical cure by the investigator but underwent a second procedure and was determined to be a failure by the Surgical Adjudication Committee.

### Safety

TEAEs occurred in 37.2% (93/250) of patients in the eravacycline group compared to 30.9% (77/249) in the meropenem group. The incidence of TEAEs reported in this and the 2 other eravacycline trials are well within the range of trials of other antibiotic therapy for cIAI [32, 33]. It is important to note that the reported TEAE rates include all events, regardless of relationship to study drug; less than half of the events reported in either treatment group were considered related to study drug.

The majority of TEAEs seen in patients who received eravacycline were gastrointestinal disorders such as nausea (n = 12), vomiting (n = 9), and diarrhea (n = 6). The full list of TEAEs that occurred in more than 2% of patients in either group can be seen in Table 9. Few events led to discontinuation of study drug in either treatment arm.

**Table 9.** Incidence of Adverse Events Occurring in >2% of Patients in Either Group: Safety Population

Medical Dictionary for Regulatory Activities Term	Eravacycline (N = 250)	Meropenem (N = 249)
Nausea	12 (4.8)	2 (0.8)
Vomiting	9 (3.6)	5 (2.0)
Infusion site phlebitis	8 (3.2)	1 (0.4)
Infusion site thrombosis	6 (2.4)	1 (0.4)
Wound infection (superficial)	7 (2.8)	4 (1.6)
Diarrhea	6 (2.4)	3 (1.2)
Anemia	3 (1.2)	6 (2.4)
Hypertension	2 (0.8)	7 (2.8)
Hypokalemia	0	6 (2.4)
Discontinued because of adverse event	4 (1.6)	4 (2.0)

Localized infusion site reactions, including infusion site phlebitis and infusion site thrombosis, were more common in eravacycline-treated patients compared to meropenem-treated patients in the study. Among these events, 3 were graded moderate in severity, and the remainder was mild. In 2 cases, the study drug was diluted into a larger volume, and in a third case, the infusion rate was decreased to manage the AE. In no case was study drug discontinued as a result of an infusion site reaction. There were 5 deaths, none of which were determined to be treatment related. The causes for these were pulmonary embolism, respiratory failure, chronic obstructive pulmonary disease, pneumonia, and cardiac arrest.

## DISCUSSION

The current clinical trial compared eravacycline (1 mg/kg IV every 12 hours) to meropenem (1 g IV every 8 hours) for the management of cIAIs. The key finding was NI of eravacycline to meropenem. These results match the findings in a previously published phase 3 study utilizing ertapenem as the comparator [34]. The particulars of this trial deserving emphasis are the microbiology encountered, response to therapy, and toxicities of eravacycline vs other tetracycline agents.

### Microbiology Encountered

The microbiology of infection and resistance encountered in this study is representative of that seen in other recent registration trials [32, 35]. The interpretation of clinical trial data for intraabdominal infection is made complex by the polymicrobial nature of these infections, the varying organ-specific processes, and the central role of source control in determining outcome [36, 37]. In the current study, polymicrobial infections were predominant, and known synergistic pairs, including gram-negative facultative and aerobic isolates and gram-positives along with *Bacteroides* and *Clostridia* species, were present in a large percentage of patients. Of note, among the 3 patients in the meropenem group with CRE isolates, 2 had polymicrobial infections.

The high rate of clinical cure among patients with *Pseudomonas aeruginosa* identified as a baseline pathogen is likely another consequence of this complexity; eravacycline has limited activity against *P. aeruginosa*, yet patients harboring this organism did equally well when treated with either eravacycline or meropenem in this study. Other trials with similar inclusion criteria have obtained the same result. We note that in the current study, of the 43 patients with *P. aeruginosa* identified as a baseline pathogen, 38 (20/22 in the eravacycline group and 18/21 in the meropenem group) had polymicrobial infections. In these cases, disruption of bacterial synergy may have contributed to the high rate of clinical cure, particularly in the eravacycline group. For patients in whom *P. aeruginosa* is suspected to be the predominant pathogen or who are at high risk of poor outcomes from this organism, such as those who are severely immunocompromised, specific therapy against *P. aeruginosa* should be considered.

As in other recently reported trials, we encountered a variety of ESBL enzyme types and CREs, and most organisms contained multiple ESBL types. Cure rates for ESBLs were uniformly high. Carbapenemase-producing pathogens were found only in patients who received meropenem, so a comparison cannot be made. To specifically address questions of the pathogenicity of ESBL-containing organisms in the presence of adequate source control, we refer to systematic reviews and observational studies that confirm improved outcomes if effective empiric treatment is provided to patients found to be infected with ESBL organisms and/or CRE [38, 39]. In ESBL bacteremia studies, cIAIs are a common source of bacteremic isolates. A recent high-quality, randomized, controlled trial confirmed these findings, with 16% of the bacteremias arising from intraabdominal sources [40]. That study found significant differences in mortality by agent used (comparing piperacillin/tazobactam with meropenem).

### Toxicities

Nausea, vomiting, and diarrhea are more common with tetracyclines than other comparable antibiotics. Tigecycline treatment in particular is associated with nausea and vomiting. A pooled analysis of 2 phase 3, double-blind trials designed to evaluate the safety and efficacy of tigecycline (vs imipenem-cilastatin) found that 24% of patients experience treatment-emergent nausea, 19% experienced vomiting, and 14% experienced diarrhea. These were significantly higher than seen in the control [41]. These problems were encountered in other randomized trials with tigecycline [42–45]. Eravacycline in this and our previous trial had comparatively low rates of TEAEs, with less than 5%, 4%, and 3% of patients experiencing nausea, vomiting, and diarrhea, respectively.

### Potential Value for Empiric and Definitive Treatment in cIAI

Eravacycline as monotherapy has demonstrated broad antimicrobial activity in both in vitro activity studies and in the 3 trials completed for cIAIs, each using a broad-spectrum carbapenem as a comparator. This agent has more potent in vitro activity than tigecycline and a substantially lessened side-effect profile. Eravacycline thus appears to be an effective agent in the therapy of cIAIs and may be an appropriate empiric choice when coverage of resistant organisms such as ESBL and CRE is desired.

## Supplementary Data

Supplementary materials are available at *Clinical Infectious Diseases* online. Consisting of data provided by the authors to benefit the reader, the posted materials are not copyedited and are the sole responsibility of the authors, so questions or comments should be addressed to the corresponding author.

ciy1029_suppl_Supplementary_Appendix

## Notes

***Acknowledgments.*** The authors thank Sergey Izmailyan and Melanie Olesky for their contributions to data retrieval and analysis.

***Financial support.*** This work was supported by Tetraphase Pharmaceuticals Inc

***Potential conflicts of interest.*** J. S. S. has received support from Tetraphase Pharmaceuticals Inc. during the production of this manuscript and has served as a consultant to Merck, Pfizer, GlaxoSmithKline, and Melinta outside of the submitted work. A. S received support from Tetraphase Pharmaceuticals Inc. during the production of this manuscript. J. G. received research funding from Tetraphase Pharmaceuticals Inc. for his role as principal investigator of this study. K. L. and L. T. are employed by Tetraphase Pharmaceuticals Inc. D. E. reports grants from Tetraphase Pharmaceuticals Inc. during the conduct of the study and grants from Merck outside of the submitted work. All authors have submitted the ICMJE Form for Disclosure of Potential Conflicts of Interest. Conflicts that the editors consider relevant to the content of the manuscript have been disclosed.
