# Supplementary material for: IGNITE4: Results of a Phase 3, Randomized, Multicenter, Prospective Trial of Eravacycline vs Meropenem in the Treatment of Complicated Intraabdominal Infections
Source: Clin Infect Dis. 2018 Dec 18;69(6):921–9. doi: 10.1093/cid/ciy1029 (PMC6735687; doi:10.1093/cid/ciy1029)
Supplement: ciy1029_suppl_Supplementary_Appendix [file ciy1029_suppl_supplementary_appendix.docx]

**Appendix**

**Complete Listing of Inclusion and Exclusion Criteria**

Patients must have met ALL of the following inclusion criteria:

1. Male or female subject hospitalized for cIAI with one of the following diagnoses:
   1. Intra-abdominal abscess: 1 or more abscesses surrounding diseased or perforated viscera (including hepatic and splenic abscesses)
   2. Gastric or intestinal perforation associated with diffuse peritonitis
   3. Peritonitis: diffuse infection of the peritoneum (but not spontaneous bacterial peritonitis associated with cirrhosis and chronic ascites)
   4. Appendicitis with perforation, peritonitis, or abscess
   5. Cholecystitis with perforation or abscess
   6. Diverticulitis with perforation, peritonitis, or abscess
   7. **Note**: Infections limited to the hollow viscus, such as simple cholecystitis and simple appendicitis, were not eligible. Ischemic bowel disease without perforation was not eligible. Acute suppurative cholangitis and acute necrotizing pancreatitis were not eligible.
2. At least 18 years of age
3. Evidence of a systemic inflammatory response with at least one of the following:
   1. Fever (oral, rectal, tympanic, or by temporal artery temperature >100.4°F/38°C) or hypothermia (temperature ≤95.9°F/35.5°C)
   2. Elevated white blood cells ([WBC] > upper limit of normal [ULN] laboratory range) or proportion of band forms of the WBC differential beyond the ULN laboratory range
   3. Increased pulse (heart rate [HR] >90 beats per minute)
   4. Increased respiratory rate (>20 breaths per minute)
4. Abdominal pain or flank pain (with or without rebound tenderness) or pain caused by cIAI that was referred to another anatomic area such as back or hip, or localized or diffuse abdominal wall rigidity, or mass, or ileus
5. Able to provide informed consent
6. Subjects must have agreed to use a highly reliable method of birth control as follows:
   1. Male subjects must have agreed to use an effective barrier method of contraception during the study and for 14 days following the last dose if sexually active with a female of childbearing potential
   2. Female subjects must not have been pregnant or nursing. For females of childbearing potential, subjects must have committed to either:
      1. Use of at least 2 medically accepted, effective methods of birth control (eg, condom, spermicidal gel, oral contraceptive, indwelling intrauterine device, hormonal implant/patch, injections, approved cervical ring, etc) during study drug administration and for 14 days following last study drug dose *OR*
      2. Sexual abstinence

And either

Subjects met all inclusion criteria for pre-operative enrollment:

1. Had a sonogram or radiographic imaging result congruent with the diagnosis of cIAI; and
2. Acute surgical or percutaneous intervention (open laparotomy, laparoscopic surgery, or percutaneous drainage of an abscess) was foreseen within 24 hours

Or

Subjects met all following inclusion criteria for intra-operative/postoperative enrollment:

1. Visual confirmation of cIAI (presence of pus within the abdominal cavity); and
2. Surgical intervention included open laparotomy, laparoscopic surgery, or percutaneous draining of an abscess; and
3. Intervention was adequate (ie, a procedure in which all communications between the GI tract and the peritoneal cavity were closed, no necrotic intestine was left, and all infected collections were drained at the procedure); and
4. Subjects who were enrolled in the trial postoperatively must have received no more than 1 dose of effective antibacterial drug therapy postoperatively before randomization

Subjects must not have met any of the following exclusion criteria:

1. Considered unlikely to survive the 6- to 8-week study period:
   1. Any rapidly-progressing disease or immediately life-threatening illness, including acute hepatic failure, respiratory failure, and septic shock
   2. Requirement for vasopressors (prior to enrollment) at therapeutic dosages (ie, dopamine >5 μg/kg/min, any dose of norepinephrine, epinephrine, or phenylephrine) to maintain a systolic blood pressure (BP) ≥90 mmHg or a mean arterial pressure ≥70 mmHg following adequate fluid resuscitation
2. Creatinine clearance (CrCL) ≤50 mL/min as estimated by the Cockcroft-Gault equation
3. Presence or possible signs of significant hepatic disease as follows:
   1. Alanine aminotransferase (ALT) or aspartate aminotransferase (AST) >5× ULN; or
   2. Total bilirubin >3× ULN, unless isolated hyperbilirubinemia was directly related to the acute process
4. Immunocompromised condition, including known human immunodeficiency virus positivity (requiring antiretroviral therapy or with cluster of differentiation 4 [CD4] count <300), autoimmune deficiency syndrome, organ (bone marrow) transplant recipients, and hematological malignancy. Immunosuppressive therapy, including use of high-dose corticosteroids (eg, >20 mg prednisone or equivalent per day for greater than 2 weeks)
5. History of moderate or severe hypersensitivity reactions to tetracyclines, carbapenems, β-lactam antibiotics, or to any of the excipients contained in the study drug formulations
6. Participation in any investigational drug or device study within 30 days prior to study entry
7. Known or suspected current central nervous system disorder that may predispose to seizures or lower seizure threshold (eg, severe cerebral arteriosclerosis, epilepsy)
8. Antibiotic-related exclusions as follows:
   1. Receipt of effective antibacterial drug therapy for cIAI for a continuous duration of >24 hours during the 72 hours preceding randomization. However, subjects with documented cIAI (ie, known baseline pathogen) who had received at least 72 hours of antibiotic therapy and who were considered treatment failures may have been enrolled. Treatment failure was defined as persistent fever and/or clinical symptoms or the development of a new intra-abdominal abscess after ≥72 hours of antibiotic therapy; or
   2. Receipt of meropenem or any other carbapenem, or tigecycline for the current infection; or
   3. Need for concomitant systemic antimicrobial agents effective in cIAI other than study drug
9. Refusal of mechanical ventilation, dialysis or hemofiltration, cardioversion or any other resuscitative measures, and drug/fluid therapy at time of consent
10. Known or suspected inflammatory bowel disease or associated visceral abscess
11. The anticipated need for systemic antibiotics for a duration of more than 14 days
12. Systemic malignancy that required chemotherapy, immunotherapy, radiation therapy, or antineoplastic therapy within the previous 3 months or which was anticipated to begin prior to the TOC Visit
13. Known at study entry to have cIAI caused by a pathogen resistant to one of the study drugs
14. Any other unstable or clinically significant concurrent medical condition (ie, class IV heart or lung disease, end stage renal disease, requiring hemodialysis, etc) that would, in the opinion of the Investigator, jeopardize the safety of a subject, impact his or her expected survival through the end of the study participation, and/or impact his or her ability to comply with the protocol

**Drug Infusion Scheme for a 24-hour Dosing Cycle**

|  | Treatment Group | |
| --- | --- | --- |
|  | Eravacycline  1.0 mg/kg IV q12h | Meropenem  1g IV q8h |
| 1^st^ Dose  60-minute IV Infusion | Eravacycline | Placebo |
| 2^nd^ Dose  30-minute IV Infusion (immediately following 1^st^ Eravacycline infusion) | Placebo | Meropenem |
| 3^rd^ Dose  30-minute IV Infusion | Placebo | Meropenem |
| 4^th^ Dose  60-minute IV Infusion | Eravacycline | Placebo |
| 5^th^ Dose  30-minute IV Infusion | Placebo | Meropenem |

Note: Infusion time could be increased to 120 minutes per IV bag to manage infusion site reactions

**Pre-Determined Definitions for Per-Pathogen Microbiological Outcomes**

Favorable

- 1. Eradication: absence of causative organism from an appropriately obtained specimen at the site of infection AT EACH TIMEPOINT
  2. Presumptive eradiation: absence of material to culture in a subject who had responded clinically to treatment

Unfavorable

- 1. Persistence: continued presence of the original pathogen in cultures from the original site of infection or blood culture obtained during or upon completion of therapy
  2. Persistence with decreased susceptibility: continued presence of the original pathogen in cultures from the original site of infection obtained during or upon completion of therapy, and the minimum inhibitory concentration (MIC) for the study drug received was at least two dilutions higher than that of the original isolate
  3. Presumed persistence: absence of material to culture in a subject who was given additional antibiotics to treat the study entry cIAI

1. Indeterminate/missing: culture not obtained

**Microbiological Specimen Collection**

Baseline intra-abdominal specimens were obtained at the time of the initial surgical intervention (or during re-intervention in the case of prior treatment failures). For those subjects who met all inclusion criteria for pre-operative enrollment, the surgical intervention occurred up to 24 hours postrandomization. Microbiological specimens collected during routine operative care prior to subject consent could have been used for study purposes. Baseline blood specimens for culture were drawn at Screening before the initiation of study drug. If more than 1 intra-abdominal or blood specimen was obtained, isolates from all specimens were reviewed for pathogen determination.

Cultures from suitable intra-abdominal specimens and blood cultures were reviewed by the Sponsor in a blinded manner prior to database lock and unblinding for final pathogen determination. A suitable intra-abdominal specimen was one that was collected from the site of infection by aspiration and/or tissue sample. Samples collected by swabs were considered suitable only if it was not possible to collect aspirated fluid or tissue samples. Samples collected from abdominal drains were not allowed and are not considered suitable samples.

Isolates cultured from suitable intra-abdominal specimens and blood cultures were initially identified at the local or regional microbiology laboratories. The identity of isolates as determined at the local or regional laboratories was verified by the central laboratory. If the local laboratory grew an isolate but the central laboratory was not able to grow the isolate, if the local laboratory grew a pathogen but the central laboratory grew only a contaminant, if isolates were lost during transportation or storage, or if there were major discrepancies between the local and central laboratories in the identification of species, the central laboratory requested that the local or regional laboratory resend the isolate. If the genus identification was the same between the local and central microbiology laboratories but the species identification was discrepant, the central laboratory identification was used. If central laboratory data were not available for an isolate, the local laboratory identification for genus and species was used. Any remaining major discrepancies in species identification between the central and local laboratories were reviewed by the Sponsor Microbiology Review Committee (MRC) in a blinded manner for final identification of the isolate.

**Procedure for Missing Data**

Missing data for the primary and secondary outcome measures of clinical response were handled as follows:

• Subjects were defined as “indeterminate” if the Investigator was not able to determine whether the subject was a clinical cure or failure. Subjects defined as indeterminate are included in the denominator for analyses in the ITT, MITT, and micro-ITT analysis populations and are, therefore, considered failures. Subjects with an indeterminate response were excluded from the CE-EOT, CE-TOC, CE-FU, ME-EOT, ME-TOC, and ME-FU Populations.

Missing data for microbiological response were handled as follows:

1. If no post-baseline source specimen was obtained and the subject had an Investigator’s assessment of clinical response, the per-pathogen microbiological response was based on the Investigator’s assessment of clinical response. A per-pathogen microbiological response was considered missing or indeterminate only if the clinical response was also missing or indeterminate.
2. Missing values for other individual data points were not imputed. Only observed values were used in data analyses and presentations.
3. Where individual data points were missing, categorical data was summarized based on reduced denominators (ie, only subjects with available data were included in the denominator).

**Definitions and Descriptions for All Analysis Populations**

Intent-to-Treat (ITT): The ITT analysis population consisted of all randomized subjects regardless of whether or not the subject received study drug. A subject was considered randomized when the Investigator or Investigator’s designee received an IWRS-generated randomization number.

Safety: The safety analysis population consisted of all randomized subjects who received any amount of study drug. All safety analyses were conducted in this population and were presented in summary tables by the treatment the subject actually received. A subject randomized to the meropenem arm who mistakenly received eravacycline was included in the eravacycline arm. A subject randomized to the eravacycline arm who mistakenly received meropenem was included in the meropenem arm if meropenem was given for the entire course of therapy and was included in the eravacycline arm if both meropenem and eravacycline were received.

MITT: The MITT population consisted of all randomized subjects who received any amount of study drug. In this study, the MITT and Safety populations were the same.

Micro-ITT: The micro-ITT population consisted of all randomized subjects in the ITT population who had at least 1 baseline bacterial pathogen against which eravacycline has in vitro antibacterial activity. As clinical breakpoints for eravacycline have not yet been determined, for the purpose of this analysis population, all baseline bacterial pathogens were considered susceptible to eravacycline.

Clinically Evaluable (CE): Subjects were included in or excluded from the CE analysis populations based on the following criteria:

1. In the MITT population: To be in included in the CE-EOT, CE-TOC, and CE-FU populations, subjects must be in the MITT population.
2. Minimal disease criteria
3. Prior antibiotic therapy: Subjects were excluded from the CE populations if they met protocol exclusion criterion 8.
4. Concomitant antibiotic therapy: Subjects who received any systemic concomitant antibiotic therapy from the first dose of study drug through the EOT Visit [CE-EOT population], the TOC Visit [CE-TOC population], and the FU Visit [CE-FU population] that was potentially effective in cIAI were excluded from the CE-EOT, CE-TOC, and CE-FU populations, respectively, with the following exceptions:
   - The subject was a clinical failure at EOT (CE-EOT population), TOC (CE-TOC population), or FU (CE-FU population) visits and received non-study antibiotics for insufficient therapeutic effect of the study drug.
   - The subject received 1 dose of a non-study antibiotic administered as prophylaxis for procedures unrelated to the ongoing infection.
   - The subject received an oral antibiotic with no systemic absorption.
5. Adequate source control: To be included in the CE-EOT, CE-TOC, and CE-FU populations, subjects must have had adequate source control. Adequate source control was determined by the SAC for those subjects with an outcome of clinical failure or with an outcome of clinical cure at the TOC or FU Visit, but underwent a second surgical procedure. All other subjects (clinical cure) were presumed to have adequate source control.
6. Study drug therapy: Subjects must have met all of the following to be included in the CE analysis populations:
   - Received the correct study drug based on the randomization assignment for the entire treatment period
   - Study personnel involved in the assessment of efficacy or monitoring efficacy data remained blinded to treatment assignment, unless a treatment limiting AE occurred which required unblinding.
   - The subject received at least 3 days of study drug.
   - The subject was at least 80% compliant with study drug.
7. Clinical outcome assessment: Subjects must have met the following to be included in the CE analysis populations:
   - For the CE-EOT analysis population:
     - Completed the Investigator’s assessment of clinical response (ie, was not deemed an indeterminate outcome) at the EOT Visit.
     - The EOT Visit occurred within 1 day from the last dose of study drug.
   - For the CE-TOC analysis population:
     - Completed the Investigator’s assessment of clinical response (ie, was not deemed an indeterminate outcome) at the TOC Visit, unless the subject was defined as a clinical failure at the EOT Visit.
     - The TOC Visit occurred on Study Days 24 to 32, unless the subject was considered to be a clinical failure based on the Investigator’s assessment at the EOT Visit.
   - For the CE-FU analysis population:
     - Completed the Investigator’s assessment of clinical response (ie, was not deemed an indeterminate outcome) at the FU Visit, unless the subject was defined as a clinical failure at the EOT or TOC Visit.
     - The FU Visit occurred on Study Days 37 to 51, unless the subject was considered to be a clinical failure based on the Investigator’s assessment at the EOT or TOC Visit.
8. Baseline or intercurrent medical events: Subjects were excluded from the CE analysis populations if the Investigator had documented in the eCRF that they meet any one of the following protocol-defined exclusion criteria at baseline (ie, prior to randomization):
   - Exclusion criterion 10: known or suspected inflammatory bowel disease or associated visceral disease
   - Exclusion criterion 12: systemic malignancy that required chemotherapy, immunotherapy, radiation therapy, or antineoplastic therapy within the previous 3 months or that was anticipated to begin prior to the TOC Visit
   - Exclusion criterion 13: Known at study entry to have cIAI caused by a pathogen(s) resistant to one of the study drugs

Microbiologically Evaluable (ME): The ME-EOT, ME-TOC, and ME-FU populations consisted of all subjects in the micro-ITT and the CE-EOT, CE-TOC, and CE-FU populations, respectively.

**Full Listing of Exclusions from the Indicated Populations**

|  | Eravacycline | Meropenem |
| --- | --- | --- |
| From CE-EOT | N=11   - Did not meet minimal disease criteria (n=1) - Received confounding non-study antibiotic (n=2) - Inadequate source control (n=2) - Did not receive at least 3 days of study drug (n=4) - Indeterminate clinical response assessment at EOT (n=8) | N=13   - Not in MITT (n=1) - Inadequate source control (n=2) - Did not receive at least 3 days of study (n=5) - Indeterminate clinical response assessment at EOT (n=13) |
| From CE-TOC | N=25   - Did not meet minimal disease criteria (n=1) - Received confounding non-study antibiotic (n=6) - Inadequate source control (n=2) - Did not receive at least 3 days of study drug (n=4) - Indeterminate clinical response assessment at TOC (n=23) - TOC visit did not occur on Study Day 24-32 (n=11) | N=19   - Not in MITT population (n=1) - Received confounding non-study antibiotic (n=5) - Inadequate source control (n=2) - Did not receive at least 3 days of study drug (n=5) - Indeterminate clinical response assessment at TOC (n=18) - TOC visit did not occur on Study Day 24-32 (n=5) |
| From CE-FU | N=21   - Did not meet minimal disease criteria (n=1) - Received confounding non-study antibiotic (n=6) - Inadequate source control (n=2) - Did not receive at least 3 days of study drug (n=4) - Indeterminate clinical response assessment at FU (n=20) - FU Visit did not occur on Study Day 37-51 (n=3) | N=19   - Not in MITT (n=1) - Received confounding non-study antibiotic (n=5) - Inadequate source control (n=2) - Did not receive at least 3 days of study drug (n=5) - Indeterminate clinical response assessment at FU (n=18) - FU Visit did not occur on Study Day 37-51 (n=5) |
| From ME-EOT | N=63  Not in Micro-ITT (n=55)  Not in CE-EOT (n=11) | N=54  Not in MITT (n=1)  Not in Micro-ITT (n=45)  Not in CE-EOT (n=13) |
| From ME-TOC | N=76  Not in Micro-ITT (n=55)  Not in CE-TOC (n=25) | N=56  Not in MITT (n=1)  Not in Micro-ITT (n=45)  Not in CE-TOC (n=19) |
| From ME-FU | N=73  Not in MITT (n=0)  Not in Micro-ITT (n=55)  Not in CE-FU (n=21) | N=58  Not in MITT (n=1)  Not in Micro-ITT (n=45)  Not in CE-FU (n=19) |

**Study Populations by Geographic Region (ITT)**

|  | Eravacycline  N=250 | Meropenem  N=250 |
| --- | --- | --- |
| European Union | 174 (69.6) | 174 (69.6) |
| Bulgaria | 52 (20.8) | 41 (16.4) |
| Czech Republic | 13 (5.2) | 16 (6.4) |
| Estonia | 20 (8.0) | 11 (4.4) |
| Hungary | 10 (4.0) | 20 (8.0) |
| Latvia | 34 (13.6) | 34 (13.6) |
| Lithuania | 22 (8.8) | 18 (7.2) |
| Romania | 23 (9.2) | 34 (13.6) |
| Non-European Union | 68 (27.2) | 72 (28.8) |
| Georgia | 8 (3.2) | 16 (6.4) |
| Russia | 21 (8.4) | 13 (5.2) |
| Ukraine | 39 (15.6) | 43 (17.2) |
| United States | 8 (3.2) | 4 (1.6) |

Baseline Microbiological Assessment (micro-ITT)

|  | Eravacycline  N=195 | Meropenem  N=205 |
| --- | --- | --- |
| Number of patients with at least 1 intra-abdominal or blood specimen | 195 (100.0) | 205 (100.0) |
| Number of patients with a baseline intra-abdominal specimen | 195 (100.0) | 205 (99.6) |
| Culture obtained by |  |  |
| Aspiration | 156 (80.0) | 170 (82.9) |
| Swab | 22 (11.3) | 19 (9.3) |
| Tissue | 115 (59.0) | 125 (61.0) |
| Growth |  |  |
| Yes | 194 (99.5) | 205 (100.0) |
| No | 1 (0.5) | 0 (0) |
| Number of patients with a sample for blood culture | 195 (100.0) | 204 (99.5) |
| Growth from at least 1 blood sample |  |  |
| Yes | 33 (16.9) | 31 (15.2) |
| No | 162 (83.1) | 173 (84.8) |

**Full Listing of Baseline Pathogens (micro-ITT)**

| Baseline Pathogen | Eravacycline | Meropenem |
| --- | --- | --- |
| Gram-negative aerobes | 141/158 (89.2) | 153/166 (92.2) |
| *Enterobacteriaceae* | 129/146 | 142/154 |
| *Citrobacter amalonaticus* | 0 | 1/1 |
| *Citrobacter braakii* | 5/5 | 0 |
| *Citrobacter freundii* | 5/7 | 1/1 |
| *Enterobacter cloacae/asburiae* | 7/7 | 5/6 |
| *Enterobacter cloacae complex* | 0 | 1/1 |
| *Escherichia coli* | 111/126 | 125/134 |
| *Klebsiella oxytoca* | 7/8 | 4/6 |
| *Klebsiella pneumoniae* | 21/21 | 23/27 |
| *Morganella morganii* | 5/5 | 3/4 |
| *Proteus mirabilis* | 1/1 | 5/6 |
| *Non-fermenting Gram-negative aerobes* | 36/38 | 28/30 |
| *Acinetobacter baumanni complex* | 5/5 | 2/2 |
| *Acinetobacter baumanni* | 0 | 2/2 |
| *Pseudomonas aeruginosa* | 18/19 | 18/20 |
| *Pseudomonas putida* | 2/2 | 0 |
| *Pseudomonas stutzeri* | 2/2 | 0 |
| *Stenotrophomonas maltophilia* | 4/5 | 3/3 |
| **Gram-positive aerobes** | 108/122 | 98/107 |
| *Enterococcus avium* | 10/11 | 9/10 |
| *Enterococcus casseliflavus* | 3/3 | 2/2 |
| *Enterococcus durans* | 1/2 | 2/2 |
| *Enterococcus faecalis* | 29/31 | 26/28 |
| *Enterococcus faecium* | 25/29 | 22/23 |
| *Enterococcus gallinarum* | 0 | 2/2 |
| *Enterococcus hirae* | 1/1 | 1/1 |
| *Enterococcus raffinosus* | 0 | 1/1 |
| *Gemella morbillorum* | 0 | 3/3 |
| *Lactobacillus delbrueckii* | 0 | 1/1 |
| *Lactobacillus fermentum* | 1/1 | 1/1 |
| *Lactobacillus gasseri/acidophilus* | 0 | 2/2 |
| *Lactococcus garviae* | 1/1 | 1/1 |
| *Lactococcus lactis* | 3/3 | 4/4 |
| *Leuconostoc lactis* | 2/2 | 0 |
| *Leuconostoc mesenteroides* | 1/1 | 1/1 |
| *Staphylococcus aureus* | 16/16 | 7/8 |
| *MSSA* | 15/15 | 7/8 |
| *MRSA* | 1/1 | 0 |
| *Streptococcus species* | 52/60 | 46/50 |
| *Streptococcus alfa-haemolyticus* | 0 | 1/1 |
| *Beta-hemolytic streptococci* | 2/2 | 4/4 |
| *Beta-hemolytic streptococcus group c* | 0 | 1/1 |
| *Streptococcus agalactiae* | 0 | 2/2 |
| *Streptococcus dysgalactiae* | 1/1 | 1/1 |
| *Streptococcus pyogenes* | 1/1 | 0 |
| *Streptococcus bovis group* | 0/1 | 2/2 |
| *Streptococcus alactolyticus* | 0 | 1/1 |
| *Streptococcus bocis group d (nonenterococci)* | 0 | 1/1 |
| *Streptococcus infantarius* | 0/1 | 0 |
| *Streptococcus viridans group* | 50/57 | 40/44 |
| *Streptococcus anginosus group* | 39/45 | 31/33 |
| *Streptococcus anginosus* | 25/29 | 21/22 |
| *Streptococcus constellatus* | 13/15 | 9/11 |
| *Streptococcus intermedius* | 2/2 | 2/3 |
| *Streptococcus mitis group* | 13/14 | 11/12 |
| *Streptococcus cristatus* | 1/1 | 0 |
| *Streptococcus gordonii* | 1/1 | 0 |
| *Streptococcus mitis/ Streptococcus oralis* | 4/5 | 5/5 |
| *Streptococcus parasanguinis* | 5/5 | 4/4 |
| *Streptococcus pneumoniae* | 1/1 | 0/1 |
| *Streptococcus sanguinis (viridans strep)* | 3/3 | 2/2 |
| *Streptococcus salivarius group* | 2/2 | 3/4 |
| *Streptococcus salivarius (viridans strep)* | 2/2 | 2/3 |
| *Streptococcus vestibularis* | 0 | 1/1 |
| **Anaerobes** | 99/110 | 104/111 |
| *Actinomyces species* | 3/3 | 3/4 |
| *Actinomyces odontolyticus* | 1/1 | 0 |
| *Actinomyces turicensis* | 2/2 | 1/1 |
| *Actinomyces viscosus* | 0 | 2/2 |
| *Bacteroides species* | 83/94 | 82/88 |
| *Bacteroides bivius* | 1/1 | 1/1 |
| *Bacteroides caccae* | 5/6 | 5/5 |
| *Bacteroides eggerthii* | 2/2 | 3/3 |
| *Bacteroides fragilis* | 33/40 | 35/38 |
| *Bacteroides intermedius* | 0 | 2/3 |
| *Bacteroides ovatus* | 19/24 | 28/28 |
| *Bacteroides salyersiae* | 0 | 1/1 |
| *Bacteroides stercoris* | 2/2 | 3/3 |
| *Bacteroides thetaiotaomicron* | 27/30 | 30/33 |
| *Bacteroides uniformis* | 14/16 | 14/14 |
| *Bacteroides vulgatus* | 27/28 | 23/23 |
| *Bifidobacterium species* | 4/4 | 3/3 |
| *Bifidobacterium adolescentis* | 0 | 1/1 |
| *Bifidobacterium bifidum* | 1/1 | 0 |
| *Bifidobacterium dentium* | 1/1 | 0 |
| *Bifidobacterium longum* | 1/1 | 1/1 |
| *Clostridium species* | 9/9 | 26/26 |
| *Clostridium cadaveris* | 0 | 1/1 |
| *Clostridium clostridiiforme* | 1/1 | 2/2 |
| *Clostridium difficile* | 0 | 1/1 |
| *Clostridium innocuum* | 0 | 3/3 |
| *Clostridium paraputrificum* | 1/1 | 1/1 |
| *Clostridium perfringens* | 7/7 | 12/12 |
| *Clostridium ramosum* | 0 | 3/3 |
| *Clostridium sordellii* | 3/3 | 3/3 |
| *Clostridium subterminale* | 0 | 1/1 |
| *Clostridium tertium* | 0 | 1/1 |
| *Collinsella species* | 2/2 | 1/1 |
| *Collinsella aerofaciens* | 2/2 | 1/1 |
| *Eggerthella species* | 1/1 | 1/1 |
| *Eggerthella lenta* | 1/1 | 1/1 |
| *Eubacterium species* | 1/1 | 2/2 |
| *Eubacterium lentum* | 0 | 1/1 |
| *Eubacterium limosum* | 1/1 | 1/1 |
| *Finegoldia species* | 2/2 | 1/1 |
| *Finegoldia magna* | 2/2 | 1/1 |
| *Flavonifractor species* | 1/1 | 0 |
| *Flavonifractor plaustii* | 1/1 | 0 |
| *Fusobacterium species* | 5/6 | 2/2 |
| *Fusobacterium mortiferum* | 1/1 | 0 |
| *Fusobacterium necrophorum* | ¾ | 0 |
| *Fusobacterium nucleatum* | 1/1 | 2/2 |
| *Megamonas species* | 2/2 | 1/1 |
| *Mobiluncus species* | 0 | 1/1 |
| *Mobiluncus mulieries* | 0 | 1/1 |
| *Odoribacter species* | 2/2 | 0 |
| *Odoribacter splanchnicus* | 2/2 | 0 |
| *Parabacteroides species* | 14/16 | 9/9 |
| *Parabacteroides (formerly Bacteroides) distasonis* | 14/16 | 9/9 |
| *Parvimonas species* | 6/7 | 5/5 |
| *Parvimonas micra (formerly Peptostreptococcus micros)* | 6/7 | 5/5 |
| *Peptostreptococcus species* | 2/2 | 0 |
| *Peptostreptococcus asaccharolyticus* | 2/2 | 0 |
| *Porphyromonas species* | 1/1 | 2/2 |
| *Porphyromonas asaccharolytica* | 1/1 | 2/2 |
| *Prevotella species* | 7/10 | 9/9 |
| *Prevotella (Bacteroides) melaningenica* | 0/1 | 1/1 |
| *Prevotella bivia* | 1/1 | 0 |
| *Prevotella buccae* | 0/1 | 2/2 |
| *Prevotella disiens* | 1/1 | 0 |
| *Prevotella intermedia* | 3/4 | 2/2 |
| *Prevotella intermedia/disiens* | 0 | 2/2 |
| *Prevotella loescheii* | 0 | 1/1 |
| *Prevotella oralis* | 1/1 | 2/2 |
| *Prevotella oris* | 1/1 | 0 |
| *Propionibacterium species* | 2/2 | 3/3 |
| *Propionibacterium acnes* | 2/2 | 3/3 |
| *Veillonella species* | 2/2 | 2/2 |
| *Veillonella parvula* | 2/2 | 2/2 |

**Monomicrobial and Polymicrobial Infections (micro-ITT)**

| Baseline Pathogen | Eravacycline  N=195 | Meropenem  N=205 |
| --- | --- | --- |
| Monomicrobial | 48 (24.6) | 72 (35.1) |
| Gram-negative aerobe | 22 | 44 |
| Gram-positive aerobe | 19 | 21 |
| Anaerobe | 7 | 7 |
| Polymicrobial | 147 (75.4) | 133 (64.9) |
| Gram-negative aerobes only | 12 | 10 |
| Gram-positive aerobes only | 5 | 1 |
| Anaerobes only | 1 | 1 |
| Gram-negative aerobe and anaerobe | 30 | 35 |
| Gram-positive aerobe and anaerobe | 4 | 8 |
| Gram-negative aerobe and Gram-positive aerobe | 26 | 18 |
| Gram-negative aerobe, Gram-positive aerobe, and anaerobe | 69 | 60 |

Clinical Response at EOT Visit

| Population | Eravacycline  (Clinical Cure/Total) | Meropenem  (Clinical Cure/Total) | Difference (95% CI) |
| --- | --- | --- | --- |
| ITT | 235/250 (94.0) | 234/250 (93.6) | 0.4 (-4.0, 4.8) |
| MITT | 235/250 (94.0) | 234/249 (94.0) | 0.0 (-4.3, 4.4) |
| Micro-ITT | 181/195 (92.8) | 193/205 (94.1) | -1.3 (-6.5, 3.7) |
| CE | 232/239 (97.1) | 234/237 (98.7) | -1.7 (-4.8, 1.1) |
| ME | 180/187 (96.3) | 193/196 (98.5) | -2.2 (-6.2, 1.2) |

Clinical Response at FU Visit

| Population | Eravacycline  (Clinical Cure/Total) | Meropenem  (Clinical Cure/Total) | Difference (95% CI) |
| --- | --- | --- | --- |
| ITT | 224/250 (89.6) | 226/250 (90.4) | -0.8 (-6.2, 4.6) |
| MITT | 224/250(89.6) | 226/249 (90.8) | -1.2 (-6.5, 4.2) |
| Micro-ITT | 170/195 (87.2) | 185/205 (90.2) | -3.1 (-9.5, 3.2) |
| CE | 220/229 (96.1) | 221/231 (95.7) | 0.4 (-3.5, 4.3) |
| ME | 168/177 (94.9) | 168/177 (94.9) | -0.9 (-5.7, 3.6) |
